# Estimating the value of democracy relative to other institutional and economic outcomes among citizens in Brazil, France, and the United States

**DOI:** 10.1073/pnas.2306168120

**Published:** 2023-11-20

**Authors:** Alicia Adserà, Andreu Arenas, Carles Boix

**Affiliations:** ^a^School of Public and International Affairs, Princeton University, Princeton, NJ 08544; ^b^IZA (Institute of Labor Economics), Bonn 53113, Germany; ^c^School of Economics, University of Barcelona, Barcelona 08034, Spain; ^d^Institut d’Economia de Barcelona, Barcelona 08034, Spain; ^e^Department of Politics, Princeton University, Princeton, NJ 08544

**Keywords:** democracy, public opinion, social welfare, measurement

## Abstract

How much do citizens value democracy? How willing are they to sacrifice their liberties and voting rights for growth, equality, or other social outcomes? We design a conjoint experiment in nationally representative surveys in Brazil, France, and the United States in which respondents choose between different societies that randomly vary in their economic outcomes (country income, income inequality, social mobility), political outcomes (democracy, public health insurance), and the level of personal income for each respondent. Our research allows us to estimate the respondents’ willingness to trade off democracy for individual income (as well as other societal attributes). We find that, on average, individuals are strongly attached to democracy and a robust welfare state. They prefer to live in a country without free democratic elections only if their individual income multiplies by at least three times and in a country without public health insurance only if their individual income more than doubles. After estimating these preferences at the individual level for all respondents, we show that, although there is an authoritarian minority in all three countries, forming a nondemocratic majority (by offering more income and/or other goods to respondents) is very unlikely. Our findings imply that, contrary to a growing discussion about the crisis of democracy, liberal democratic values remain substantially robust in high and middle income democracies.

## Significance

Growing political polarization and the rise of populist parties in various parts of the world have raised substantial concerns about the attachment of citizens to democracy and the durability of liberal institutions. To estimate the value that citizens give to democracy relative to income and other features of society, we field survey experiments in Brazil, France, and the United States asking respondents to choose among different alternative societies. Although we find that all countries harbor a minority with authoritarian preferences, having democratic institutions strongly trumps all other considerations in determining the optimal society for an overwhelming majority of citizens. It is therefore difficult to conclude that democracy is fundamentally in crisis, at least at the public opinion level.

The stability and ultimate survival of democracies rely on the willingness of political actors—both citizens and political officials—to subject themselves to elections and accept the possibility of losing them (1–3). According to conventional measures of popular support for democracy, most countries meet this requirement. Most opinion surveys show nominal adherence to democracy to be overwhelming across the world. Over 90% of respondents prefer democracy to any other political alternative, such as military rule or technocratic management, in almost all countries included in comprehensive opinion polls such as the World Value Survey and the Latinobarometer (4–6). Even among groups where support for democracy has allegedly declined in recent years [for example, among young cohorts (7)], that erosion has been marginal (8). Standard opinion surveys face, however, two important limitations. First, respondents may hide their nondemocratic opinions out of social desirability bias. Second, and more crucially, they are asked to state their preferences for democracy without having to bear any costs or confront any tradeoffs associated with their opinions and without engaging in any comparison with potentially different societal and institutional arrangements. Perhaps for these reasons, the evidence on the level of citizen support needed to achieve democratic stability is mixed (8–13).

In this paper, we design a conjoint experiment to elicit the preferences of citizens about democracy relative to other fundamental features of society. More specifically, we ask survey participants to rate and choose between pairs of hypothetical societies. For each pair, we randomly assign the presence or absence of free elections. In addition, we vary the societies offered to respondents along three dimensions: the respondents’ individual economic conditions (defined by their individual income); aggregate economic conditions in terms of the average income in that society, the prevailing level of income inequality, and the prevalent mechanisms that determine personal and social mobility (either effort or connections); and, finally, the presence of a robust welfare state (in the form of public health insurance). This setup enables us to explore public support for democracy in three ways. First, because we randomize the assignment of individual income (as well as other economic parameters), we can quantify the monetary value of democracy, that is, the price citizens demand to prefer a society without free elections. Second, we can leverage our results to address existing research on whether public opinion perceives the existence of a potential tradeoff between democracy and prosperity or the provision of public goods (14, 15). Third, we employ our estimates of the individual preferences toward different features of society to find the conditions under which there could be a majority of voters that, after weighing the set of alternative outcomes or institutions they would enjoy under a nondemocratic solution, would turn against democracy (or any other collective outcome).

We run our conjoint experiment in three countries (Brazil, France, and the United States) that provide us with different levels of economic development and political institutions, and where authoritarian and antiglobalist politicians have achieved high levels of popularity (Bolsonaro, Le Pen, and Trump). The inclusion of the United States also allows us to compare our work to recent conjoint analysis on the attitudes of ideologically polarized voters toward candidates exhibiting different levels of attachment to democracy (16, 17).

In all three countries, we find strong preferences for living in a society with democratic elections. On average, our respondents need to experience a very large increase in their individual income (around three times the average monthly income of the country) to give up on free elections. This is a much larger “compensation” than the one they demand to give up either public health insurance or a meritocratic society and certainly much larger than the value of living in an egalitarian society.

The level of support for democracy across and within countries is not uniform. Across countries, Americans value democracy the most. French and Brazilian respondents value it less, even though the difference is small in relative terms. In turn, each country includes a minority of nondemocratic respondents. Still, the median respondent values democracy highly. Indeed, we find it rather difficult to put together an alternative bundle of institutions and economic and social outcomes that can persuade enough pro-democracy respondents to join the nondemocratic minority.

Both our design and findings make several contributions to the study of the foundations of democracy and, more generally, other social institutions. First, we improve on opinion surveys employed to study the level of democratic commitment among citizens by minimizing the latter’s potential social desirability bias. Second, we measure the preferences of citizens toward democracy in a setup that arguably approaches real-world choices more closely than previous studies: By including a rich array of economic and institutional parameters characterizing the individual welfare of respondents and the societal context in which they would live, we force respondents to confront potential tradeoffs in the choice of economic and political institutions.^*^ Third, we offer a method to calculate, in a direct way, our respondents’ willingness to pay for (or, in other words, the price of) democracy (or for any other social institution, for that matter). This method allows us to tackle an important topic of discussion in the literature: The extent to which citizens may accept authoritarian regimes for the sake of growth, less corruption, or even economic redistribution.

## The Value of Democracy

We assess the value of democracy by fielding a conjoint experiment in Brazil, France, and the United States, through online surveys administered to 2,000 participants in each country in the fall and winter of 2021–2022 by the firm Netquest. The Princeton University Institutional Review Board approved the survey. The samples were designed to be nationally representative of age, gender, education, and region.

In the conjoint experiment, each individual was presented with seven pairs of alternative societies and was then asked both to choose one among the pair and to rate each alternative on a scale from 0 to 10. This generated about 28,000 observations per country (number of respondents × 7 × 2).

The conjoint experiment contained six attributes that, without possibly being exhaustive given the limits of any conjoint (18), aimed at describing the broad political and economic traits that define a given society and the respondent’s position in it: individual monthly income, average monthly income, presence or absence of free elections, a public health insurance system, underlying general social norms defining social advancement, and level of income inequality. Table 1 describes the values over which these different attributes were randomized. The treatment on individual income, country income, and inequality received by each respondent was based on the average country income at the time of the survey, that is, €3,000 in France, 3,000 Brazilian Reals in Brazil, and $6,000 in the United States. Thus, for example, French respondents were asked to choose between societies with a country income of €4,500, €3,000, or €2,400. *Materials and Methods* discusses the conjoint design and the survey procedure in detail.

**Table 1.** Randomized treatments in conjoint experiment

Attribute	Number of treatments	Content of treatments
1. Individual income	5	Income equivalent to 1.25, 1.1, 1, 0.9, and 0.8 times the average monthly income in each country at the time of survey
2. Country income	3	Income equivalent to 1.5, 1, and 0.8 times the average monthly income in each country at the time of survey
3. Political institutions	2	“People choose the national government through free elections” vs. “There are no free elections to choose the national government”
4. Health system	2	“There is a public health system paid by an income tax” vs. “Health is not covered by a public health system”
5. Getting ahead	2	“Personal connections matter more than effort” vs. “Effort is more important than personal connections”
6. Inequality	2	“The maximum and minimum income of the country are 2 and 0.5 times the country’s average income” vs. “The maximum and minimum income of the country are 4 and 0.25 times the country’s average income”

We examine the value of democracy using three measures: its average marginal component effect; the willingness to pay; and the weight respondents give to democracy relative to other attributes.

### Average Marginal Component Effect (AMCE).

The AMCE of each attribute represents the average change in the probability of choosing a hypothetical society (or, alternatively, in its rating) associated with a switch in the level of that particular societal attribute (19).

**Fig. 1.**
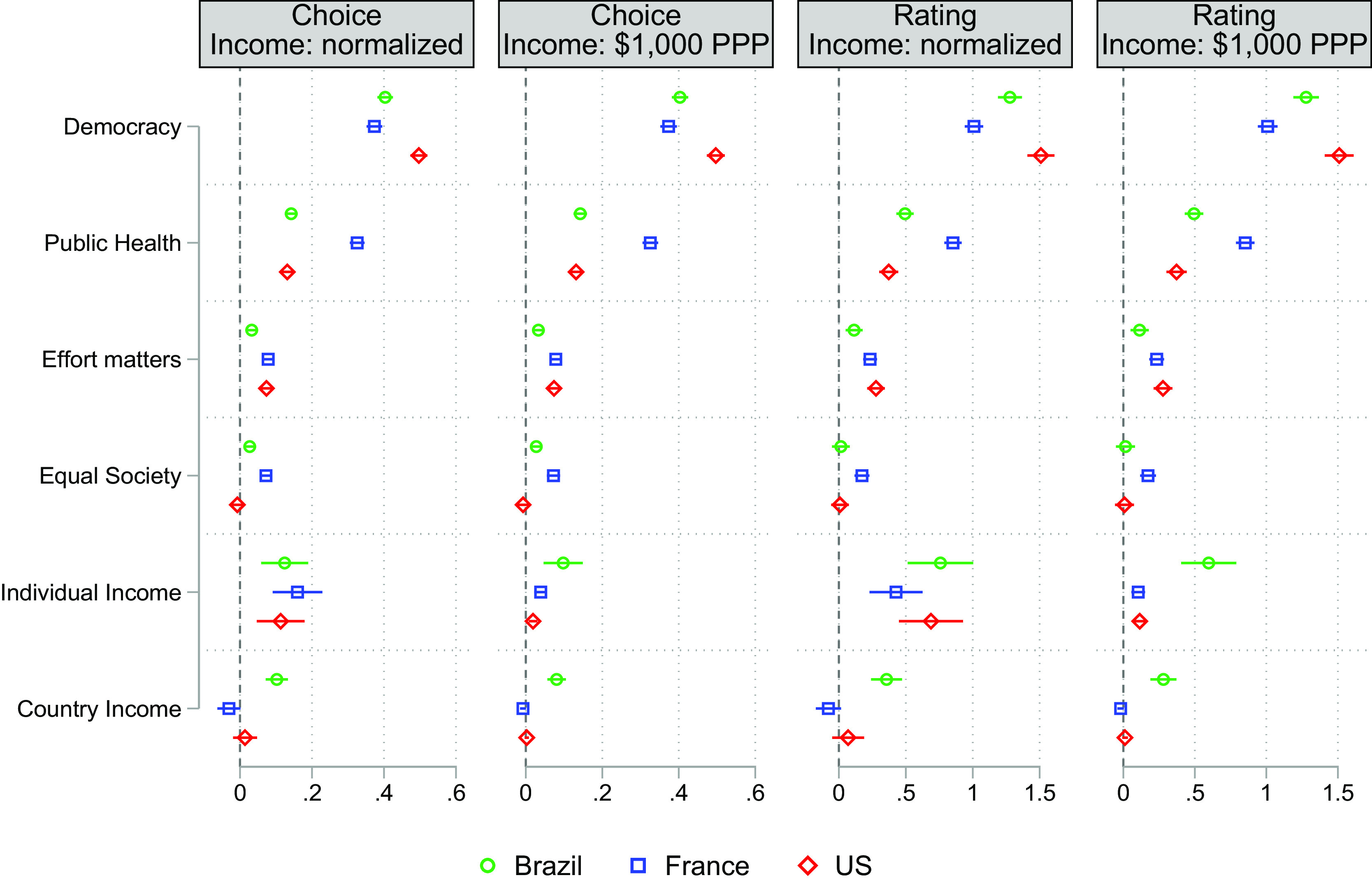
Average marginal component effects. Note: Normalized income has been normalized by the country average, i.e., one unit increase means an increase equivalent to 100% of the country average income, which is 1,270PPP$ in Brazil, 4,100PPP$ in France, and 6,000PPP$ in the United States, based on OECD data (https://www.oecd-ilibrary.org/finance-and-investment/purchasing-power-parities-ppp/indicator/english_1290ee5a-en). All specifications include individual-specific pair fixed effects and control for the alternatives’ position (Left–Right). CIs from SEs clustered by survey participant.

Fig. 1 displays estimates of AMCEs by country, for both choices (Panels 1 and 2) and ratings (Panels 3 and 4). All the alternatives’ attributes are binary, with the exception of country and individual income, which vary over three and five different values, respectively. To facilitate the presentation of the results, we treat the latter two as continuous. In the first and third panels, we normalize the income variables by their country averages, which means that one unit of income is equal to €3,000 in France, to 3,000 Brazilian Reals in Brazil, and to $6,000 in the United States. In the second and fourth panels, income is in thousands of purchasing power parity dollars (PPP$).

The preference for a democratic society appears as the strongest of all the attributes in our conjoint. Everything else equal, the probability of choosing a society increases by 40% among French and Brazilian respondents if there are free elections, rising to above 50% among US respondents. There is one additional attribute for which French respondents show a similar attachment: public health insurance. Their likelihood of selecting a society with that welfare state institution rises by around 35%. The effect is much smaller in Brazil and the United States, at around 15%. The impact of other collective traits (effort and equality) pales in comparison. Employing rating values, we obtained slightly attenuated effects, but the magnitudes and cross-country patterns are similar.

When we normalize income with respect to the country mean, the AMCEs of individual income are rather similar across countries—at around 0.1 (Panel 1). However, given different per capita incomes across countries, these estimates represent very different quantities. Those differences become apparent when we report the coefficients for a $1,000 (in PPP terms) income increase in the second and fourth panels.^†^ An increase of $1,000 (in PPP terms) has a much larger effect in Brazil, where the AMCE is around 0.1, than in France or the United States, where the effect is only slightly above 0. To put it differently, a similar raise in absolute terms of the individual income makes a given society more attractive to Brazilians than to French or US respondents. We expand on the mechanisms behind this finding in the next subsection, when discussing Table 2.

**Table 2.** Willingness to Pay (using rating estimations)

	WTP for normalized income			WTP and income in $1,000 PPP		
	(1) Brazil	(2) France	(3) US	(4) Brazil	(5) France	(6) US
Democracy	168.407^***^ (28.39)	236.373^***^ (56.52)	219.415^***^ (39.57)	2.141^***^ (0.36)	9.714^***^ (2.32)	13.165^***^ (2.37)
Public health	65.069^***^ (11.42)	199.626^***^ (48.48)	54.076^***^ (11.02)	0.827^***^ (0.15)	8.204^***^ (1.99)	3.245^***^ (0.66)
Effort matters	14.959^**^ (4.91)	54.558^***^ (14.51)	40.281^***^ (8.12)	0.190^**^ (0.06)	2.242^***^ (0.60)	2.417^***^ (0.49)
More Equal	1.946	40.369^***^	1.172	0.025	1.659^***^	0.070
Society	(4.50)	(11.84)	(4.90)	(0.06)	(0.49)	(0.29)
10% increase	4.693^***^	-1.836	1.000	0.060^***^	-0.075	0.060
in Country income	(1.08)	(1.22)	(0.91)	(0.01)	(0.05)	(0.05)
N	26,740	23,702	22,736	26,740	23,702	22,736
Measure of preference	Rating	Rating	Rating	Rating	Rating	Rating

Entries are ratios of AMCEs. ACMEs estimated with individual-specific pair fixed effects and position controls. Delta method SEs in parenthesis. ^*^(P<0.10), ^**^(P<0.05), ^***^(P<0.01). Columns 1–3: WTP as a percentage of the average country income. Columns 4–6: WTP in thousands of PPP$.

### Individual Willingness to Pay (WTP).

The WTP (calculated as the ratio of the AMCE of a given attribute to the AMCE of individual income) is the additional income required, on average, to persuade respondents to give up on a particular attribute, e.g., democracy, as a feature of the society of their choice, or, in other words, the monetary price of that attribute. (See *Materials and Methods* for the computational procedure.)

Table 2 reports the increase in individual income needed to make individuals indifferent between a democratic society and a nondemocratic society in Row 1. We also extend the computation of the willingness to pay to all the other attributes: health care protection, meritocracy, income equality, and a higher country income. For space considerations, we report here the WTP estimates based on the results obtained from asking individuals to rate the alternative societies they encounter.^‡^

Columns 1–3 report the WTP derived from using a normalized individual income: The numbers display the percentage increase in income needed. The standard errors of all these coefficients’ ratios are computed using the delta method. On average, respondents in all three countries appear to have a similar underlying willingness to pay for democracy. Their individual income would have to triple to give up free elections—with French respondents preferring a country without free democratic elections only if their individual income more than tripled and Brazilians being marginally more tolerant toward nondemocratic institutions.

The commitment to democracy appears as particularly salient once we compare it to other societal features. French respondents value public health almost as much as democracy: They would only prefer a country without public health insurance if their individual income tripled. Otherwise, democracy’s WTP is much larger than any other WTP’s estimates. Among Brazilians and Americans, their income would have to increase by 65 and 54%, respectively, to give up a public health system. In turn, to live in a country where connections, rather than effort, mattered, Brazilians, French, and US citizens would need to experience an increase of 15, 55, and 40% of their individual income respectively. Preferences over country income and income inequality are both more subdued and more heterogeneous across countries. French respondents would give up having an equal society (which here means lowering the maximum-to-minimum income ratio from 16 to 4, that is, a 75% decrease) if their income rose by 40%. Brazilians and Americans care, but only very marginally, for a more equal society. Finally, to accept a poorer country (specifically, 10% poorer), Brazilians would need to experience close to a 5% increase in their individual income. The estimated WTP for a higher country income is not statistically significant in either France or the United States.

Columns 4–6 estimate the WTP relying on the AMCE based on a fixed increase (of $1,000 in PPP terms) in individual income across all countries. Numbers are expressed in unit increases of that absolute amount of income. Differences across countries now become starker. Brazilians appear willing to surrender democracy if their monthly income increases by $2,141. French respondents would need more than four times that amount to give up on democracy. Americans would only become indifferent after a raise of $13,165.^§^

We interpret the different WTPs we obtain for an increase in normalized income and a raise in fixed income as evidence for the interplay of two mechanisms. On the one hand, the WTP estimates in Columns 1–3 indicate that the overall preference for democracy (in relationship to an identical proportional income shift in each country) is similar across all respondents. On the other hand, the estimates in Columns 4–6 suggest that, due to the decreasing marginal utility of income, the average respondent in a country with a higher average income needs more income than the respondent in a poorer country to give up any other valuable social attribute (democracy in this case).^¶^

Cross-national differences are also substantial in the dimensions of public health and meritocracy (when using a change in absolute income). An average Brazilian is willing to give up public health insurance for $827. The price of public health insurance is much higher in the United States and, particularly, in France. French respondents require an extra $8,204 to give up a public health system. The average American respondent would for $3,245. Effort has a similar price in France and the United States. Finally, French respondents continue to value equality much more strongly than the other two countries.

### The Relative Weight of Democracy.

The willingness to pay for democracy indicates the value of democracy relative to the income that respondents may earn in a nondemocratic society. Nevertheless, the choice of democratic institutions is embedded in a broader setup defined by the possibility of having other desirable attributes, such as public health or a merit-based society, besides personal income. Accordingly, we now shift from calculating the WTP (based on income) to estimating the weight $\omega_{f}$ that respondents place on a specific economic and institutional feature *f* (for example, democracy) that they encounter when choosing between different societal bundles in terms of all the remaining desirable attributes that they may forsake for that feature *f* (such free and fair elections). (*Materials and Methods* reports the procedure to compute $\omega_{f}$.)

Table 3 reproduces the estimated weights using the coefficients from ratings. (For the estimates of the weights $\omega_{f}$ based on choice, see *SI Appendix*, Table S5.) The weight that respondents place on democracy (to select a society) represents 42.4%, 36.4%, and 51.6% of all the attributes in Brazil, France, and the United States respectively. With the exception of France, the relative weight of democracy is much higher than the second most important factor: public health insurance. The choice between having a public health system or not has a weight of 30.7% in France, 16.4% in Brazil, and 12.7% in the United States. The weight of individual income ranges between 15 and 25%.^#^ The remaining factors have a smaller weight in shaping the decisions of respondents: between 3 and 9% for effort and less for equality. Only Brazilians give some weight to country income. In short, democracy continues to be central to voters even when they consider it relative to all the other features.^‖^

**Table 3.** Attributes’ weights (rating)

	(1) Brazil	(2) France	(3) US
Democracy	0.424^***^ (0.023)	0.364^***^ (0.017)	0.516^***^ (0.028)
Public health	0.164^***^ (0.012)	0.307^***^ (0.016)	0.127^***^ (0.013)
Effort matters	0.038^***^ (0.010)	0.084^***^ (0.009)	0.095^***^ (0.010)
More equal society	0.005 (0.011)	0.062^***^ (0.010)	0.003 (0.012)
Country income	0.118^***^	0.028	0.024
(100% increase)	(0.018)	(0.017)	(0.020)
Individual income	0.252^***^	0.154^***^	0.235^***^
(100% Increase)	(0.032)	(0.032)	(0.033)
N	26,740	23,702	22,736
Measure of preference	Rating	Rating	Rating

Entries are the absolute value of the AMCE (of the attribute) over the sum of the absolute value of all AMCEs. AMCEs specification includes individual-specific pair fixed effects and position controls. Delta method SEs in parenthesis. ^*^(P<0.10), ^**^(P<0.05), ^***^(P<0.01).

## Individual Preferences and Nondemocratic Coalitions

So far, we have estimated country-average preferences for democracy (and the remaining attributes). We turn now to recover the underlying distribution of individual preferences for democracy within each country by employing the fourteen observations we have per respondent. In the previous subsection, we estimated the absolute weight $\omega_{f}$ for each feature. Here, instead, we compute the individual-level estimates of the real value of the weight $w_{d}$ for democracy. (For the computation procedure, see *Materials and Methods*.) This allows us to generate a distribution that goes from −1 to 1 and that, therefore, measures both the relevance given to democracy (or any other feature) in the choice of the society under consideration and the direction of the weight given to democracy (with a negative weight implying that having a democracy makes the respondent less willing to choose and rate favorably that particular society).

**Fig. 2.**
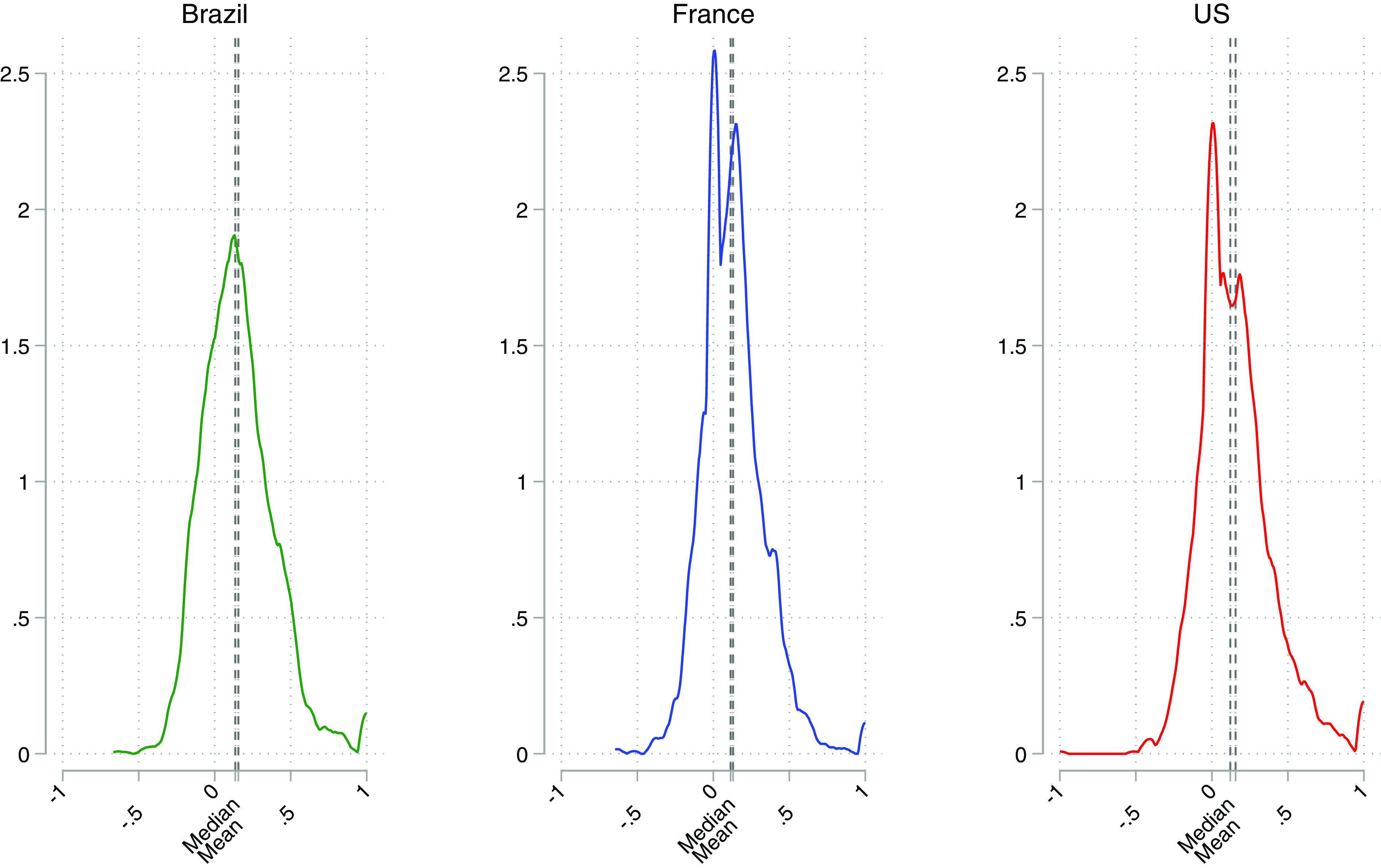
Density distribution of individual-level weights of democracy across countries. Preference measurement: Ratings.

Fig. 2 plots the density distribution of the estimated individual weights $w_{d}$ for each country separately. All the plots report the estimates derived from rating alternative societies.^**^ Individual values over democracy are relatively spread out in all countries—approximating a normal distribution. Both the median and the mean are around 0.2 and a clear majority—at least three out of four respondents—weigh democracy positively. Most respondents to the left of 0 have small negative relative weights. The average weight of the respondent in the twentieth percentile (in the distribution of respondents from lowest to highest weight) is −0.03. The average weight of the individual in the tenth percentile is −0.11. Only about 3% of all respondents have a relative weight below −0.20 or less. In short, most of the members of the nondemocratic minority we have identified oppose democracy rather weakly.^††^

### Nondemocratic Coalitions.

We turn now to employ the individual-level marginal component effect of the conjoint attributes (IMCEs) to determine the conditions under which there can be a majority of voters that, after weighing the set of alternative outcomes or institutions they would enjoy under a nondemocratic solution, would oppose democracy (or support its demise). More generally, we can use the procedure to calculate the predicted level of public support for various alternative societies.

More precisely, we can compute how each respondent would evaluate democratic and nondemocratic societies as we manipulate their remaining societal attributes. We do this by first considering two identical societies in all dimensions except political regime, that is, we compare a democracy and a nondemocracy, and estimating the percentage of our respondents choosing one over the other. We then improve the nondemocratic alternative relative to the society with democracy by manipulating one attribute at a time and recomputing the proportion “voting” for each one after each manipulation. As we show shortly, democracy turns out to be a substantially resilient institution in the cases we study: A nonauthoritarian majority only emerges, if at all, after we add up a lot of valuable attributes to the nondemocratic option.

**Fig. 3.**
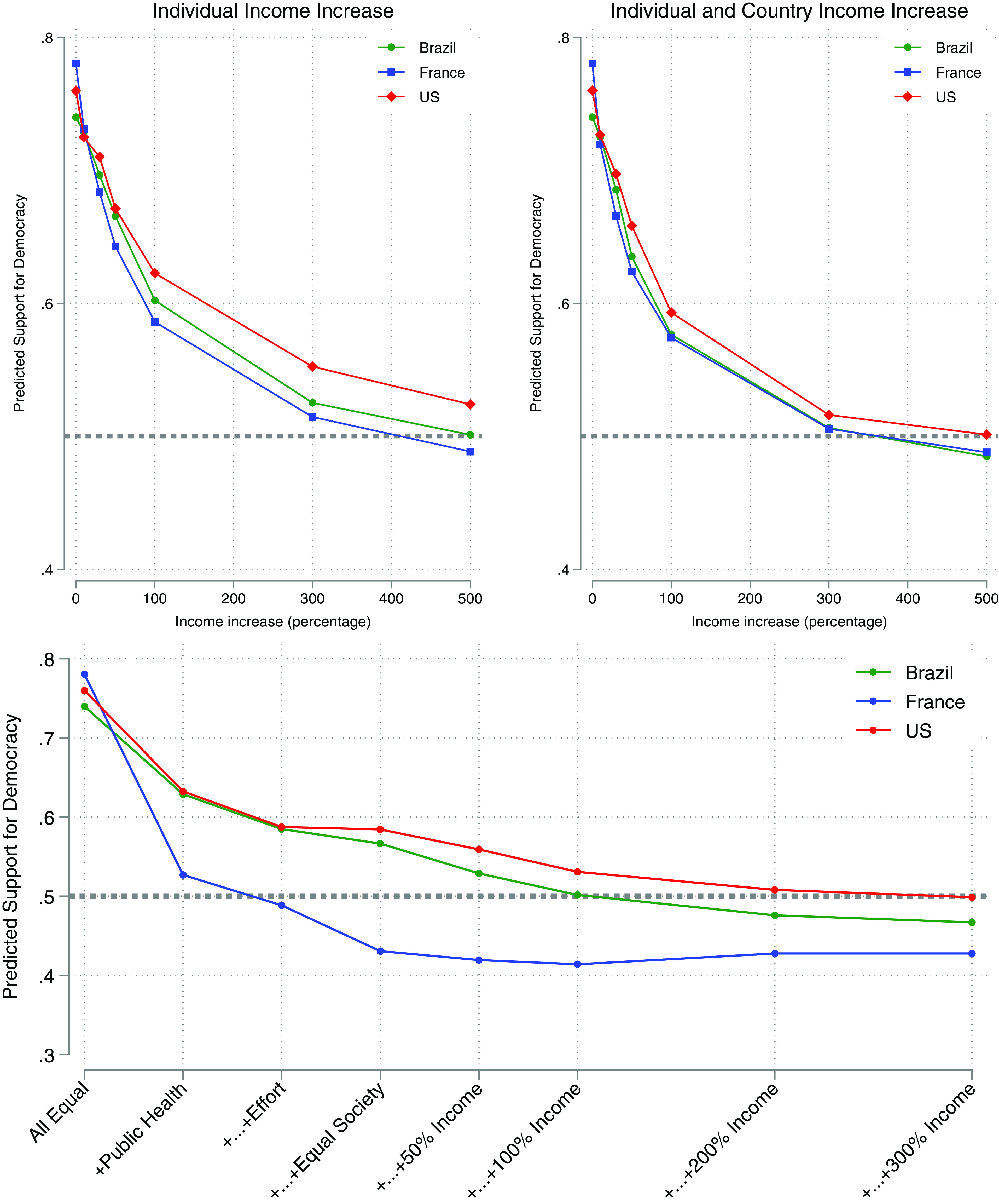
The construction of nondemocratic coalitions. Note: monthly income increase for both country and individual income. All other features held constant unless otherwise stated. Preference measurement: ratings.

We start by studying whether democracy can be defeated with economic growth alone. In the *Top-Left* panel of Fig. 3, we gradually increase individual incomes in the nondemocratic alternative and plot the corresponding decline in support for democracy. Holding everything else constant, democracy has a wide initial level of support at around 75%. As individual incomes rise in nondemocratic alternatives (while remaining unchanged in the democratic option), that majority shrinks. It does, however, at a rather slow rate. Only extremely high increases in individual income lead to a majority of respondents preferring a nondemocratic society. Note, however, that this is a *partial equilibrium* exercise because it is not possible to increase everybody’s income by 500% without increasing the country’s income. Accordingly, the top right panel engages in the same exercise now manipulating both country and individual incomes. Support for democracy declines at a slightly faster rate. Still, only very high increases in income (about 400% in Brazil and France, about 500% in the United States) result in a majority of respondents preferring a nondemocratic society.

In the *Bottom* panel of Fig. 3, we repeat the same exercise, now switching first the institutional attributes to make the nondemocratic society more attractive. As before, the leftmost point in the graph reports the support for democracy (at around 75%) when comparing two societies that differ only in the use of free elections to choose their government. We then compare a democracy without public health to a nondemocracy with public health (while maintaining all other conditions identical across both societies). Support for the democratic option falls to slightly above 50% in France. Once we switch the meritocracy attribute and compare a democratic society working on connections and with no public health to a nondemocracy with a publicly provided health system and governed by the principle of merit, support for democracy drops further. A slight majority of French respondents go authoritarian.^‡‡^ By contrast, a majority of Brazilian and American respondents still prefer the alternative with free elections. They continue to do so even after the democratic option includes a wider distribution of income and personal income doubles under authoritarianism. Among Brazilian respondents, a nondemocratic society only dominates a democratic society once it has public health, meritocracy, economic equality, and a country and individual income that are 200% higher than the one under democracy. Finally, the United States crosses the point to an authoritarian majority only when the nondemocratic society dominates the democratic society in public health, meritocracy, economic equality, and when country and individual income are four times higher than in the authoritarian alternative.

## Discussion

To determine the value of democracy and the extent to which citizens may be willing to sacrifice their liberties and voting rights for growth, equality, or just their ideological commitments, we have designed a conjoint experiment in which respondents have to choose (almost as if they acted under a veil of ignorance) between pairs of hypothetical societies that differed randomly on several dimensions: private outcomes (individual income), economic aggregate outcomes (level of economic development, income inequality, social mobility), and political outcomes (democracy, public health insurance).

Our paper contributes to the study of the foundations of democratic institutions along different dimensions. First, our experimental design reduces the potential social desirability bias that threatens most opinion surveys on democratic preferences. Second, we embed the choice of democracy within a set of alternative societies characterized by an array of economic and institutional parameters that arguably shape the individual welfare of respondents. This decision setup is different from recent survey designs that measure democratic preferences in the framework of candidate-choice conjoint experiments (16, 17). Our design forces respondents to confront tradeoffs in the selection of key economic and political institutions, opening up the possibility of examining the extent to which citizens may accept authoritarian regimes for the sake of growth, less corruption, and even welfare redistribution. Finally, and relatedly, we offer a set of computational procedures to calculate the “price” of democracy (or any other societal feature). We believe that these procedures can be put to use profitably to study more general questions in the fields of social choice and institutional design.

Our findings suggest that support for democracy is quite robust, at least in middle- and high-income societies. In Brazil, France, and the United States, having free elections emerges as the feature that respondents value the most in choosing among alternative pairs of societies (as well as rating them). According to our estimations, having free elections (or not) accounts for almost half of the decision that respondents make in their choices. This is much larger than any other variable. The weight respondents place on public health insurance, which is the second most preferred attribute on average, ranges (depending on the country) from one eighth and one third of all the features that respondents take into consideration.

Democracy is not just the most important factor for respondents when making a decision. It is also intensely desired vis-à-vis other attributes. To forgo democracy, the average respondent would have to be paid at least three times his or her income. And, even though we uncover a (weakly) nondemocratic minority that fluctuates around one fifth of our respondents, we observe the presence of a strong prodemocratic supermajority that would need a substantial monetary compensation to give up on free elections.^§§^${}^{,}$^¶¶^

Our results speak to recent work on the basis of democracy in at least two ways. First, even though current candidate-choice conjoint experiments on democratic preferences and our own design are not directly comparable because the objects to be selected by their respondents (politicians versus societies) differ in nature, we see both studies as complementary (rather than necessarily opposed). Candidate-choice experiments have so far exposed voters to minor (and sometimes ambiguous) democratic violations (16, 17, 22).^##^ Because these studies rely on a low “threshold” to define a particular practice as nondemocratic, they find a relatively high toleration of voters toward candidates endorsing democratic violations. By contrast, we advance a much more demanding definition of democratic breakdowns. In our analysis, a country qualifies as a democracy if it has free elections. As a result, a much smaller proportion of the electorate condones the lack of democracy. Putting the two approaches together, it appears that the extent to which citizens acquiesce to any democratic violations varies as a function of the magnitude of the latter.

Second, and directly related to our previous point, the structure of democratic preferences we uncover (in particular, the resistance of citizens to major violations of democracy) questions main tenets of the “democratic backsliding” literature. In a seminal article on the potential decay of contemporary democracies, (26) points out that the old, classical forms of democratic breakdown (mainly coups and blatant electoral fraud) have given way, in recent decades, to processes of dedemocratization by stealth, notably through executive aggrandizement and the strategic manipulation of elections and electoral institutions. Even though the causes and the mechanisms of this process of democratic erosion often remain imprecise in the literature, backsliding theorists generally agree on the following story (27, 28). Increasing societal polarization (either caused by cultural or economic changes or perhaps simply spurred by populist politicians) generates a reservoir of intensely partisan voters that political incumbents can employ to peel off democratic checks and norms one at a time until their electoral advantage becomes insurmountable. This story does not seem to be borne, however, by our findings. A strong majority of our respondents seem unwilling to live in a society where leaders do not respect the fundamental tenets of democracy. This, in turn, should make it hard for political incumbents to violate central democratic norms and institutions while sustaining their initial electoral coalition, at least in middle- and high-income democracies. Our results are in fact in line with a growing empirical literature that does not find much evidence pointing to the decline and death of democracy today (29–31).

This does not mean, however, that democracies cannot collapse. As pointed out before, our study uncovers, in each of the countries under study, a nondemocratic minority that, if well-organized, could act to frustrate and suppress widespread support for democracy. But rather than being linked to a backsliding story, this result takes us back to a long tradition in democratization theory that stresses social conflict, the role of economic and political elites and organizations (in relationship to that conflict), and the use of violence to explain authoritarian politics (32–34). It is there where we may still need to look at to understand the mechanics of democratic breakdowns.

## Materials and Methods

### Conjoint Experiment.

In the conjoint experiment, each individual was presented with seven pairs of alternative societies and was then asked both to choose one among the pair and to rate each alternative on a scale from 0 to 10 (*SI Appendix*, Fig. S1 includes the “Survey Instructions” given to each respondent. *SI Appendix*, Fig. S2 reproduces an example of the vignette faced by respondents. At several points at the beginning of the survey experiment, respondents were told to assume constant prices across scenarios). This generated about 28,000 observations per country (number of respondents × 7 × 2). At the start of the survey, we stated that participation was voluntary and that information would be published in an anonymous form. Respondents were also given the contact information of Princeton IRB before they were asked to consent.

The conjoint experiment contained six attributes that, without possibly being exhaustive given the limits of any conjoint (18), aimed at describing the broad political and economic traits (summarized in Table 1) that define a given society and the respondent’s position in it:

1. Individual monthly income, which we randomized over five variants, each one equivalent to 1.25, 1.1, 1, 0.9, and 0.8 times the average monthly income in each country at the time of the survey.
2. Average monthly income of society, for which we considered the three variants of 1.5, 1, and 0.8 times the average monthly income in country at the time of the survey.
3. Political institutions. The treatment was randomized over two alternatives. In the first one, the individual was informed that “people choose the national government through free elections.” In the second one, the respondent learned that “there are no free elections to choose the national government” [On the scholarly literature that examines the use of the term “free elections” as a key element (and a shortcut) to define democracy, see refs. 35 and 36).
4. A public health insurance system, introduced to convey the presence of a comprehensive welfare state. Respondents were either informed that “there is a public health system paid by an income tax” or told that “health is not covered by a public health system.”
5. Our fifth treatment described the underlying general social norms defining personal advancement. In the first scenario, respondents were informed that either “personal connections matter more than effort to get ahead.” In the second one, we learned that “effort is more important than personal connections to get ahead.” This distinction around the presence or absence of meritocratic principles has been consistently validated as a crucial dimension along which respondents across countries characterize how their own society and economy operate. See, for example, ref. 37.
6. Our final treatment described the extent of inequality in each hypothetical society, randomized over two possibilities: a relatively equal society where “the maximum income in the country is (2 times the average monthly income) and the minimum is (0.5 times that the average monthly income)” and a relatively unequal society where “the maximum income in the country is (4 times that the average monthly income) and the minimum is (0.25 times that the average monthly income).” Notice that because the average monthly income can take three values, the income inequality treatment could take any of six variants. The magnitude of this treatment, that we label “more equal society” entails a reduction in the ratio of the maximum to the minimum income in society of 75% (i.e., from 16 to 4).

The surveys also included questions to gather information about the demographic attributes (gender, education, income, religion), political preferences (left/right, trade, immigration, technology, beliefs about causes of economic success), and psychological traits (dark triad—Machiavellism, narcissism, psychopathy) of respondents (*SI Appendix*, Tables S1–S3 report covariate balance tests for all the treatments by social category, revealing no difference between treatments, as expected given their random assignment across alternatives and individuals). In addition, the surveys contained debriefing questions on the reasons respondents had to choose different alternatives. In our analysis, we exclude respondents who completed the survey in less than 10 min—this results in a drop of about 10% of our sample. Results are, however, robust to their inclusion (*SI Appendix*, Fig. S3 examines the consistency between choices and ratings by survey length deciles. For respondents who completed the survey quickly, results are slightly more inconsistent).

### Estimation of AMCEs.

We estimate AMCEs with linear regressions of the outcome variables (either a dummy for choosing an alternative or not, or the rating of the alternative) on the attributes of the alternative society: democratic elections, public health insurance, social advancement based on effort, economic equality, country income, and individual income. The data are at the participant-by-hypothetical society level. The estimation of the AMCE takes the following form:

$$Y_{\mathrm{ijk}}=\alpha_{\mathrm{ij}}+\sum_{l=1}^{l=6}\beta^{l}Attribute_{\mathrm{ijk}}^{l}+\gamma X_{\mathrm{ijk}}+v_{\mathrm{ijk}},$$

where we regress the outcome Y (either choice or rating) by individual i of the alternative k in the pair j on the six attributes’ ($Attribute^{1}$ (democracy) to $Attribute^{6}$ (country income) characterizing the hypothetical alternative. We introduce individual-by-order fixed effects $\alpha_{\mathrm{ij}}$ (i.e., pair fixed effects, to exploit variation only within alternatives that were shown simultaneously) and control for whether the alternative was shown in the left or right side of the screen ($\gamma X_{\mathrm{ijk}}$). SEs are clustered at the survey participant level (In *SI Appendix*, Figs. S4 and S5 compare the estimates with and without individual-by-order fixed effects. The inclusion of these controls barely changes point estimates. The only exception concerns the country and individual income attributes. When exploiting only within-pair variation, the income variables have a slightly larger effect, presumably because side-by-side differences are more salient than across pairs).

In the model for choice, the dependent variable which takes a value of either 0 or 1 depending on whether the society is chosen by the respondent. In the model of rating, where every participant rates fourteen hypothetical societies presented in seven pairs, the outcome ranges from 0 to 10.

### Estimation of Willingness to Pay.

To compute the WTP, we take the ratio of the AMCE of democracy to the AMCE of individual income. When we normalize income with respect to the country mean, because normalized income is measured in hundreds per cent, the ratio alone would give us the WTP in hundreds per cent. Therefore, we multiply it by 100 to obtain the WTP measured in percentages. For democratic elections, we define:

$${\hat{\mathrm{WTP}}}_{Normalized\;income}^{Democratic\;Elections}=\frac{{\hat{\beta }}^{Democratic\;Elections}}{{\hat{\beta }}_{\mathrm{normalized}}^{Individual\;Income}}\times 100.$$

When computing the WTP in $ 1,000 PPP, we use the same expression, but without multiplying it by 100:

$${\hat{\mathrm{WTP}}}_{\$1000\;\text{PPP income}}^{Democratic\;Elections}=\frac{{\hat{\beta }}^{Democratic\;Elections}}{{\hat{\beta }}_{\$1000\;\text{PPP}}^{Individual\;Income}}.$$

### Estimation of Absolute and Real Weights.

We compute the *absolute* weight or value placed by respondents on each attribute relative to all the remaining attributes n as a normalized weight $\omega_{f}$ or the ratio of the absolute value of the AMCE of attribute f (that is, $|\beta_{f}|$) over the sum of the absolute values of all AMCEs [We follow here the estimation model proposed by Graham and Svolik (16)]. Formally,

[1] $$\omega_{f}=\frac{|\beta_{f}|}{\left[\sum_{n=1}^{N}|\beta_{n}|\right]}.$$

In turn, we compute the *real* weight $w_{d}$ as the ratio of the real value of the marginal component effect of attribute f over the sum of the absolute values of all marginal component effects. Formally,

[2] $$w_{d}=\frac{\beta_{f}}{\left[\sum_{n=1}^{N}|\beta_{n}|\right]}.$$

## Supplementary Material

Appendix 01 (PDF)

## Data Availability

Anonymized Survey data have been deposited in Harvard Dataverse https://doi.org/10.7910/DVN/RTUFHH (38).

## Footnotes

^*^In using the term of “tradeoff,” we do not imply that there is actually one between different outcomes (for, e.g., between democracy and growth). We simply stress the fact that respondents, by being offered alternative societies varying on different dimensions, may have to “sacrifice” certain goods for others.

^†^One dollar was equivalent to 0.7 euro and to 2.7 Brazilian real at the time the surveys were fielded.

^‡^We follow the same practice throughout the rest of the paper. WTP estimates based on forcing individuals to choose societies are be found in *SI Appendix*, Table S4.

^§^A battery of tests do not reveal loss aversion in the structure of individual preferences over income (20), making a linear estimation reasonably valid to approximate the preferences of respondents. Results are available from the authors.

^¶^We find a similar effect at the individual level: High-income individuals need higher income raises than low-income respondents do to give up democratic institutions. In the section “Individual Preferences and Nondemocratic Coalitions,” we calculate the marginal component effect of the conjoint attributes at the individual level (IMCEs). We then report the IMCEs for democracy by income level of our respondents (as well as several other covariates recorded in the surveys) in *SI Appendix*.

^#^For both individual and country income, we have set the value of the attribute at a 100% increase of income.

^‖^As in any other conjoint study, the interpretation of our results could be affected by the possibility that respondents associate (the effects of) an attribute of the treatment (i.e., democracy) with other attributes in the treatment. As we develop in *SI Appendix*, section 4, we believe this concern is minimized for two reasons. First, in line with our experimental design, we interpret the preference for democracy as net of its effects on all the other treatments we include. Second, we show that the difference between the AMCE for democracy and the AMCE that would encompass all the indirect effects of democracy is likely to be small.

^**^Because we only have fourteen observations per individual, the individual-level estimates of democracy weights are noisy—with varying levels of noise across individuals. *SI Appendix*, Fig. S6 displays their density distribution using inverse-variance weighting, which takes into account the individual-level standard errors of the estimated weights, and shows a very similar pattern. *SI Appendix*, Fig. S7 displays the density distribution based on choices.

^††^*SI Appendix*, Fig. S8 displays the percentiles of the distribution of the individual-level weight on democracy by country.

^‡‡^In France, a nondemocracy with 200% or 300% more income has slightly less support than a nondemocracy with 100% more income because 1) our simulation is based on individual-level estimates and 2) about 40% of French respondents dislike living in a country with higher income (including themselves being richer). Although they may prefer nondemocratic alternatives that outperform democracies in public health, meritocracy, and economic equality, a small fraction among them switch again to back up the democratic alternative when we increase the country income in a nondemocratic alternative.

^§§^The size of the nondemocratic minority we identify is in line with the findings of several surveys on public support for democracy. For example, ref. 21 reports that, in a set of opinion polls conducted in the United States between 2010 and 2017 using items originally designed for Latin America, about a quarter of US respondents agreed that a military coup would be justified under some circumstances. Although high, the United States’ share of nondemocratic respondents was smaller than in almost all other American countries, where support for a coup ranged from a third to a half of all the respondents. Notice that we measure our respondents’ willingness to pay for a minimalist, electoral democracy. Eliciting a more expansive definition of democracy (with civil rights, etc.) would have probably increased their WTP.

^¶¶^In *SI Appendix*, Tables S6–S8, we report the strength of democratic commitments across some social categories such as age, gender, education, psychological traits, and policy preferences. The nondemocratic minority is larger in size among younger cohorts, less educated individuals, and antiglobalization sectors. Its members score higher in the psychological traits of Machiavellism and psychopathy.

^##^For studies relying on survey experiments (that do not rely on conjoints), see, among others, refs. 23–25.
