# Supplementary material for: Estimating the value of democracy relative to other institutional and economic outcomes among citizens in Brazil, France, and the United States
Source: Proc Natl Acad Sci U S A. 2023 Nov 20;120(48):e2306168120. doi: 10.1073/pnas.2306168120 (PMC10691337; doi:10.1073/pnas.2306168120)
Supplement: Supplementary file 1 — Appendix 01 (PDF) [file pnas.2306168120.sapp.pdf]

Supporting Information for

Estimating tradeoffs between democratic representation and other institutional and economic outcomes among citizens in Brazil, France, and the United States

Alicia Adsera, Andreu Arenas, Carles Boix.
Corresponding author, Carles Boix: cboix@princeton.edu

This PDF file includes:

Figs. S1 to S8
Tables S1 to S8

1. Conjoint Procedure. Survey Instructions and Conjoint Example.

Fig. S1. Survey Instructions

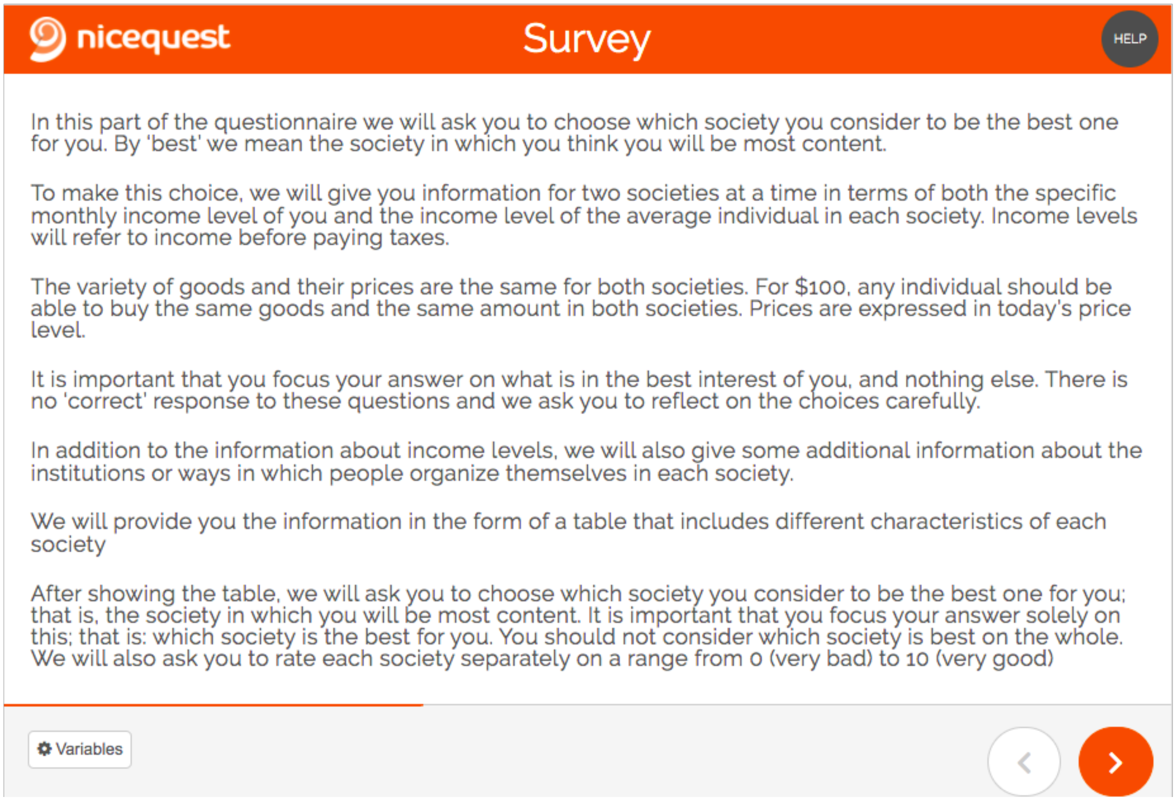

Fig. S2. Example of Conjoint Faced by Respondent

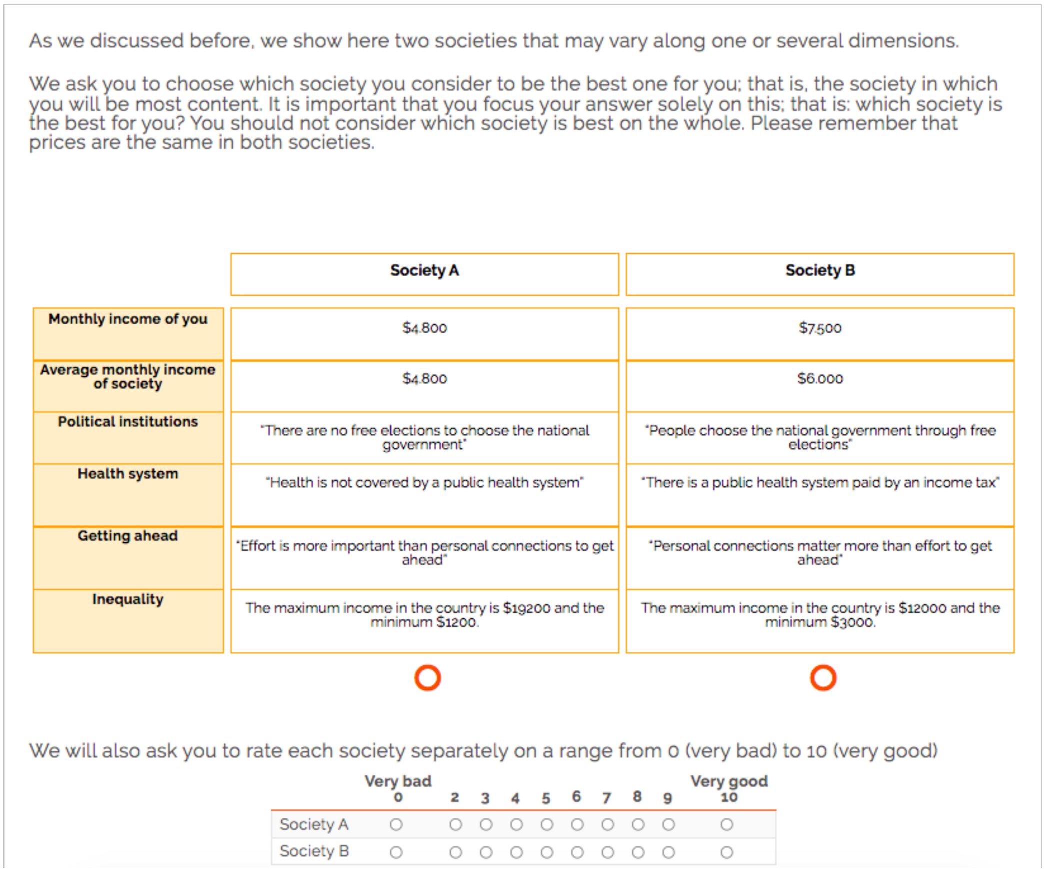

As we discussed before, we show here two societies that may vary along one or several dimensions.

We ask you to choose which society you consider to be the best one for you; that is, the society in which you will be most content. It is important that you focus your answer solely on this; that is: which society is the best for you? You should not consider which society is best on the whole. Please remember that prices are the same in both societies.

	Society A	Society B
**Monthly income of you**	$4.800	$7.500
**Average monthly income of society**	$4.800	$6.000
**Political institutions**	"There are no free elections to choose the national government"	"People choose the national government through free elections"
**Health system**	"Health is not covered by a public health system"	"There is a public health system paid by an income tax"
**Getting ahead**	"Effort is more important than personal connections to get ahead"	"Personal connections matter more than effort to get ahead"
**Inequality**	The maximum income in the country is $19200 and the minimum $1200.	The maximum income in the country is $12000 and the minimum $3000.

We will also ask you to rate each society separately on a range from 0 (very bad) to 10 (very good)

	Very bad 0	2	3	4	5	6	7	8	9	Very good 10
Society A	○	○	○	○	○	○	○	○	○	○
Society B	○	○	○	○	○	○	○	○	○	○

Fig. S3. Examination of Consistency between Choices and Ratings by Survey Length

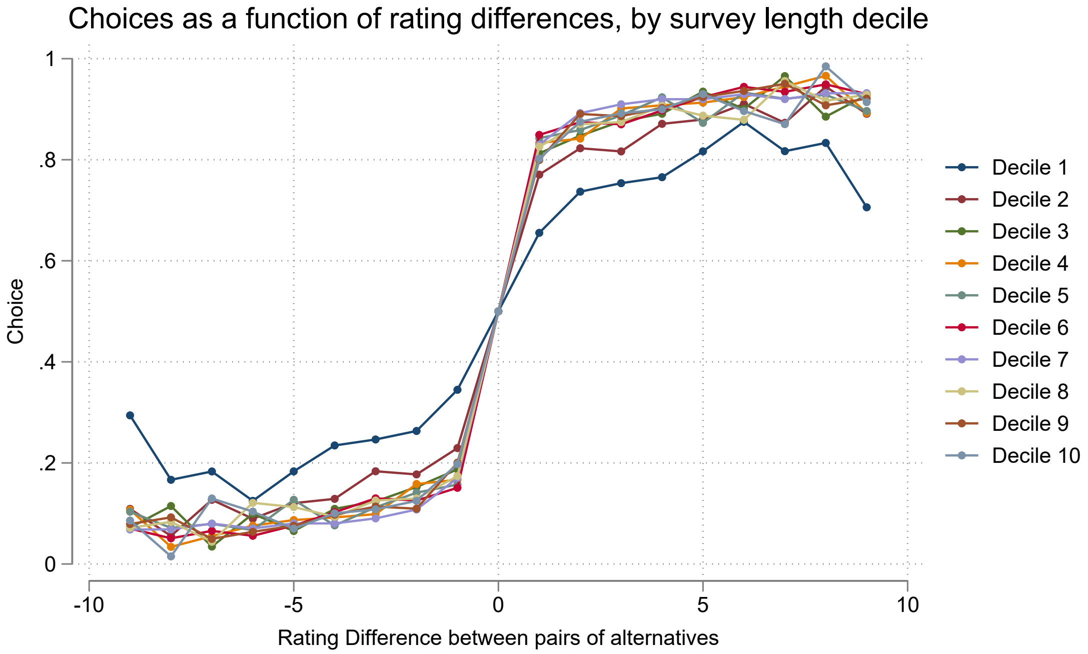

Inconsistencies: positive rating difference and no choice, or negative rating difference and choice.
Decile 1 = lowest length, Decile 10, highest length.

2. Results. Figures

Fig. S4. AMCEs with and without Pair Fixed Effects (normalized income)

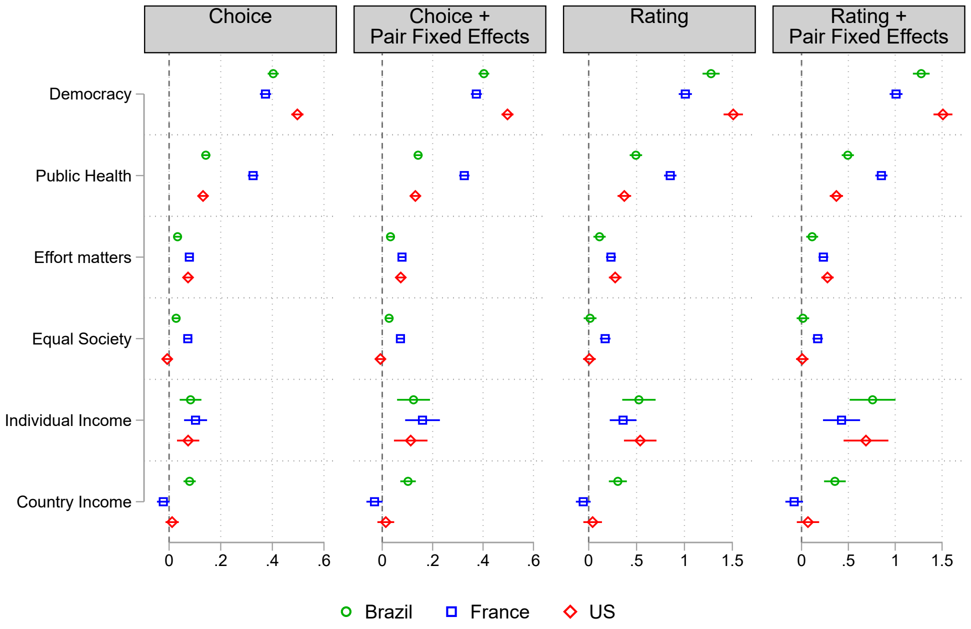

Note: Normalized income has been normalized by the country average, i.e. one unit increase means an increase equivalent to 100% of the country average income, which is 1,270PPP$ in Brazil, 4,100PPP$ in France, and 6,000PPP$ in the United States, based on OECD data (link to source). All specifications control for the alternatives' position (left/right) and order (1st to 7th screen). Pair fixed effects specifications include individual-specific pair fixed effects. Confidence intervals from standard errors clustered by survey participant.

Fig. S5. AMCEs with and without Pair Fixed Effects (income in 1,000 of PPP$)

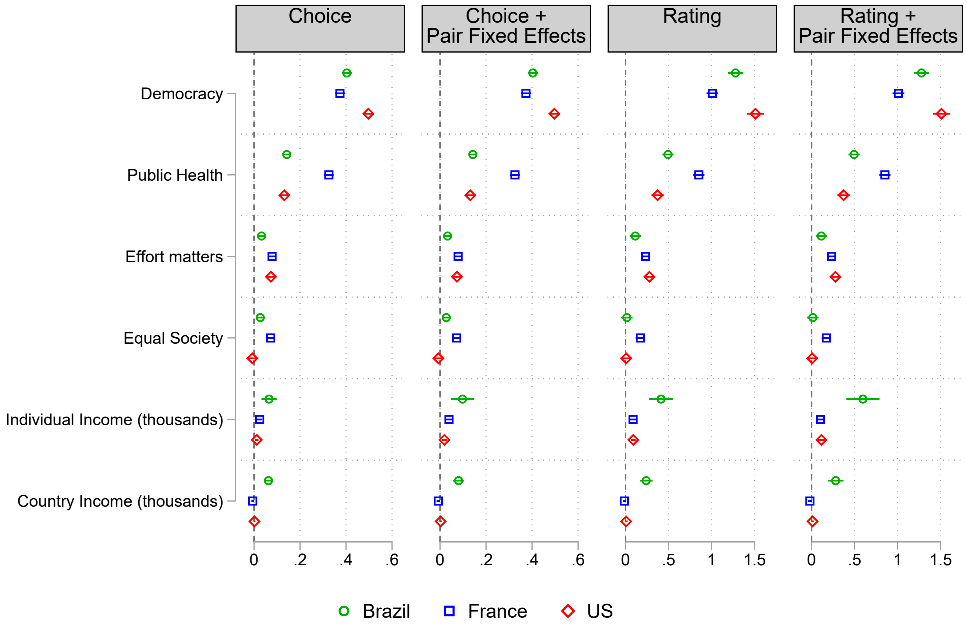

Fig. S6. Density Distribution of Individual-level Weights of Democracy across Countries. Pref. Measurement: Ratings. Inverse-variance Weighted Results.

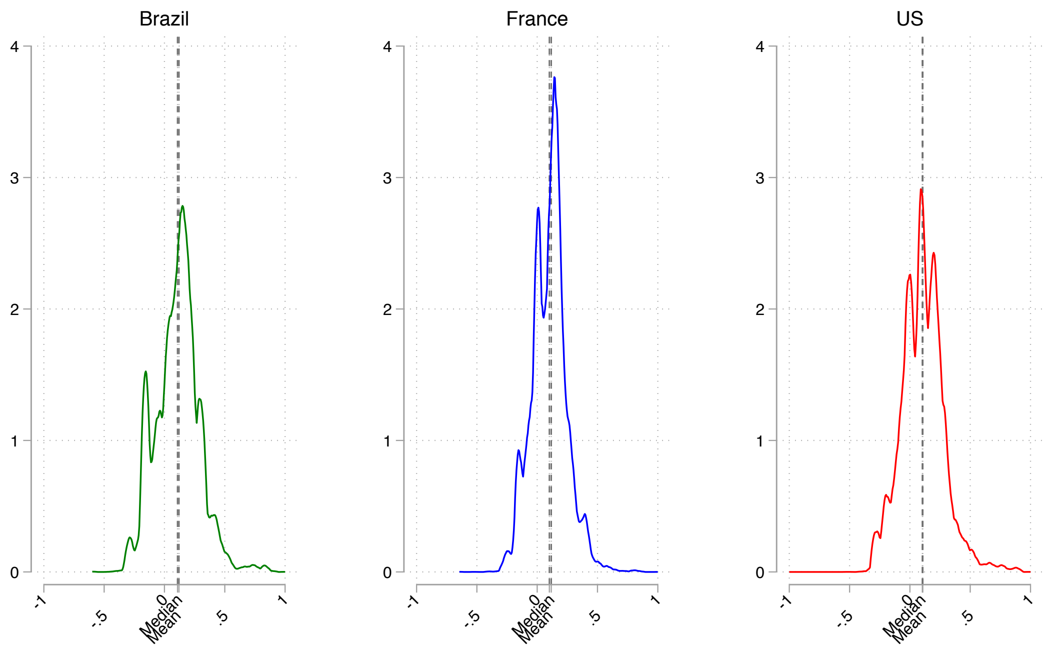

Note: for observations without defined variance, we input the minimal defined variance in the sample.

Fig. S7. Density Distribution of Individual-level Weights of Democracy across Countries. Pref. Measurement: Choice.

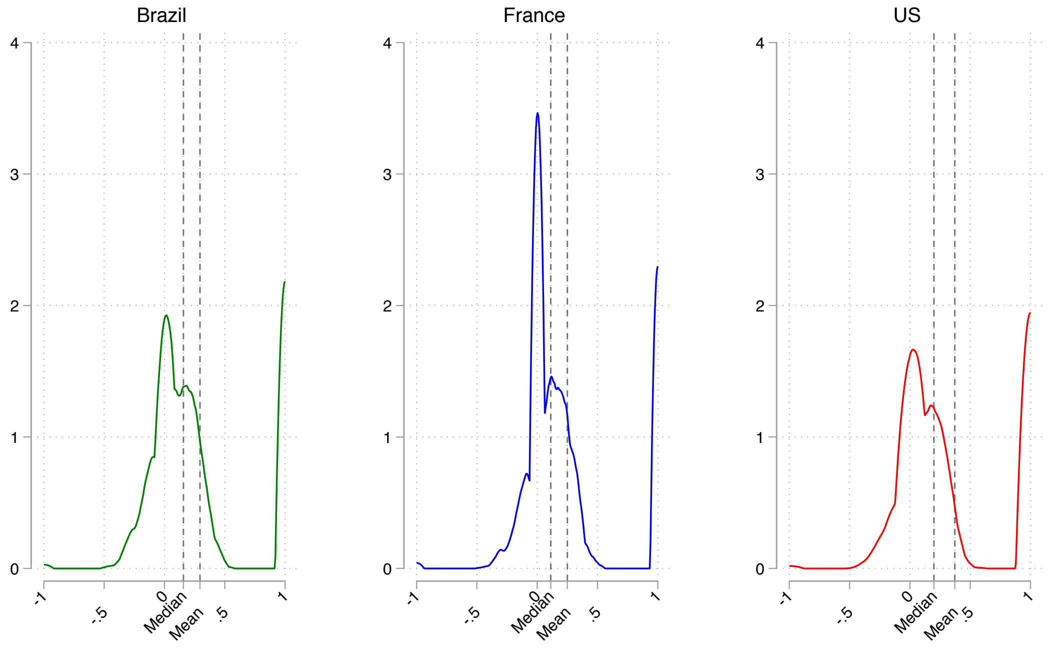

Fig. S8. Percentiles of individual-level Weights of Democracy across Countries. Pref measurement: ratings.

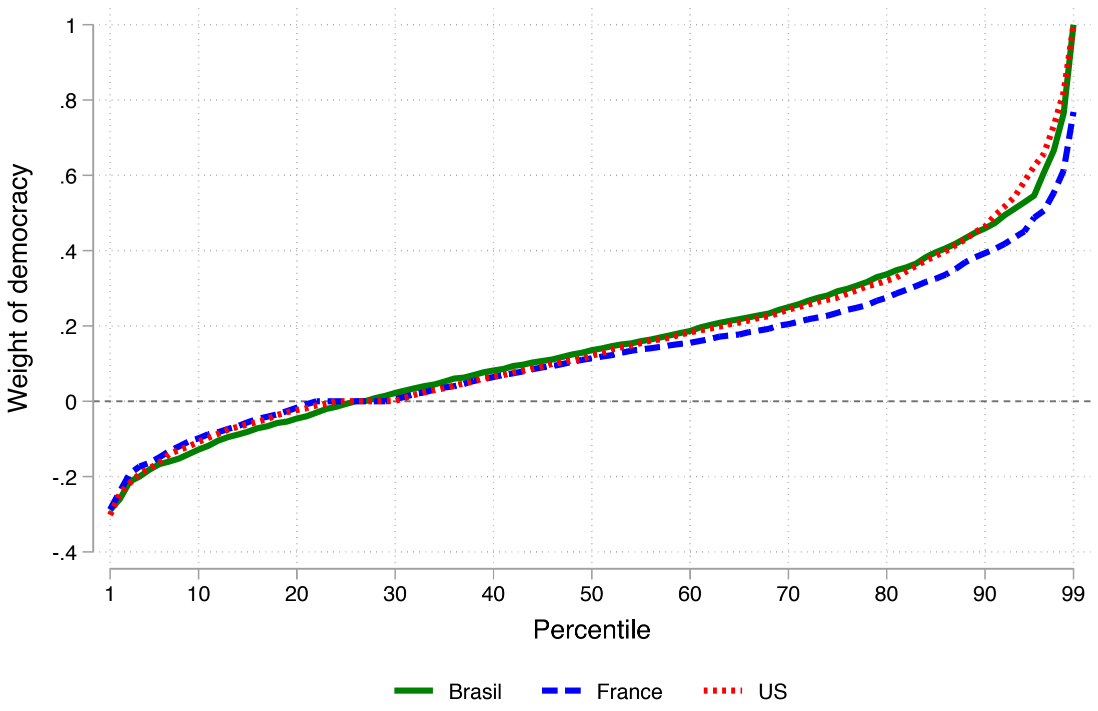

3. Results. Tables

Table S1. Covariate Balance Test for All Treatments, By Social Category. Brazil

	(1)	(2)	(3)	(4)	(5)	(6)
	E[Covariate | Treatment = 1] - E[Covariate | Treatment = 0]					
Variable	Democracy	Public Health	Effort	Equal Society	High I. Income	High C. Income
Fraction with High Income	-0.000	0.000	0.000	-0.000	0.001	0.004
	(0.006)	(0.006)	(0.006)	(0.006)	(0.007)	(0.008)
Fraction with Very High Income	0.000	0.000	0.000	0.000	0.001	0.005
	(0.004)	(0.004)	(0.004)	(0.004)	(0.005)	(0.005)
HH Wealth (0-10 index)	-0.000	0.000	-0.000	-0.000	0.005	-0.035
	(0.030)	(0.030)	(0.030)	(0.030)	(0.033)	(0.037)
Parental Wealth (0-10 index)	-0.000	0.000	0.000	-0.000	0.014	-0.034
	(0.031)	(0.031)	(0.031)	(0.031)	(0.034)	(0.038)
Fraction with college	0.000	-0.000	0.000	0.000	-0.000	0.001
	(0.005)	(0.005)	(0.005)	(0.005)	(0.005)	(0.006)
Fraction with high school or less	0.000	-0.000	-0.000	-0.000	0.002	-0.000
	(0.006)	(0.006)	(0.006)	(0.006)	(0.007)	(0.007)
Age (continuous)	-0.000	-0.000	-0.000	0.000	0.034	-0.190
	(0.188)	(0.188)	(0.188)	(0.188)	(0.210)	(0.235)
Fraction of females	0.000	-0.000	-0.000	0.000	-0.000	-0.009
	(0.006)	(0.006)	(0.006)	(0.006)	(0.007)	(0.008)
Machiavellianism (standardized index)	-0.000	0.000	-0.000	0.000	0.008	0.006
	(0.012)	(0.012)	(0.012)	(0.012)	(0.014)	(0.015)
Narcissism (standardized index)	0.000	0.000	-0.000	-0.000	-0.002	-0.004
	(0.012)	(0.012)	(0.012)	(0.012)	(0.013)	(0.015)
Psychopathy (standardized index)	-0.000	-0.000	0.000	0.000	0.001	-0.015
	(0.012)	(0.012)	(0.012)	(0.012)	(0.013)	(0.015)
Right wing (1-10 scale)	-0.000	0.000	-0.000	0.000	0.012	0.000
	(0.032)	(0.032)	(0.032)	(0.032)	(0.035)	(0.040)
Pro-trade (1-5 scale)	-0.000	0.000	-0.000	-0.000	0.008	0.011
	(0.010)	(0.010)	(0.010)	(0.010)	(0.012)	(0.013)
Pro-immigration (1-5 scale)	-0.000	0.000	0.000	-0.000	0.004	0.002
	(0.011)	(0.011)	(0.011)	(0.011)	(0.012)	(0.014)
Pro-tech. change (1-5 scale)	-0.000	0.000	0.000	-0.000	0.000	0.007
	(0.010)	(0.010)	(0.010)	(0.010)	(0.011)	(0.012)
Status $>$ Concrete (Pr. Component)	0.000	-0.000	-0.000	-0.000	-0.001	-0.013
	(0.014)	(0.014)	(0.014)	(0.014)	(0.016)	(0.017)
Observations	26,740	26,740	26,740	26,740	26,740	26,740

Robust standard errors in parenthesis. * $p < 0.10$, ** $p < 0.05$, *** $p < 0.01$. High I. Income: individual income treatment above the country monthly average. High C. Income: country income treatment above the country average. High income: household income above the country monthly average (3000 reals/euro, or 6000 USD). Very high income: monthly household income above 5000 reals/euro 5000, or 9000 USD). Machavellianism, narcissism and psychopathy are standardized to have mean zero and standard deviation of one, from the sum of scores in 5 questions. Status $>$ Concrete is the 2nd principal component of the responses to debriefing questions about income motivations in answering the conjoint, which features positive weights for status motivations for income and negative weights for material motivations for income.

Table S2. Covariate Balance Test for All Treatments, By Social Category. France

	(1)	(2)	(3)	(4)	(5)	(6)
	E[Covariate | Treatment = 1] - E[Covariate | Treatment = 0]					
Variable	Democracy	Public Health	Effort	Equal Society	High I. Income	High C. Income
Fraction with High Income	-0.000	-0.000	-0.000	-0.000	0.000	-0.005
	(0.006)	(0.006)	(0.006)	(0.006)	(0.007)	(0.008)
Fraction with Very High Income	0.000	-0.000	0.000	-0.000	0.000	0.000
	(0.003)	(0.003)	(0.003)	(0.003)	(0.003)	(0.003)
HH Wealth (0-10 index)	-0.000	-0.000	0.000	0.000	-0.007	-0.036
	(0.025)	(0.025)	(0.025)	(0.025)	(0.028)	(0.031)
Parental Wealth (0-10 index)	-0.000	0.000	-0.000	0.000	-0.013	-0.005
	(0.027)	(0.027)	(0.027)	(0.027)	(0.031)	(0.034)
Fraction with college	0.000	0.000	0.000	-0.000	0.001	-0.007
	(0.006)	(0.006)	(0.006)	(0.006)	(0.007)	(0.008)
Fraction with high school or less	-0.000	0.000	-0.000	0.000	-0.000	0.002
	(0.005)	(0.005)	(0.005)	(0.005)	(0.005)	(0.006)
Age (continuous)	0.000	-0.000	0.000	0.000	0.037	-0.093
	(0.226)	(0.226)	(0.226)	(0.226)	(0.253)	(0.282)
Fraction of females	0.000	-0.000	0.000	0.000	-0.002	-0.002
	(0.006)	(0.006)	(0.006)	(0.006)	(0.007)	(0.008)
Machiavellianism (standardized index)	0.000	0.000	0.000	-0.000	-0.002	-0.007
	(0.013)	(0.013)	(0.013)	(0.013)	(0.014)	(0.016)
Narcissism (standardized index)	0.000	-0.000	0.000	-0.000	-0.008	-0.001
	(0.012)	(0.012)	(0.012)	(0.012)	(0.014)	(0.015)
Psychopathy (standardized index)	0.000	0.000	0.000	0.000	-0.005	0.003
	(0.011)	(0.011)	(0.011)	(0.011)	(0.013)	(0.014)
Right wing (1-10 scale)	0.000	0.000	0.000	0.000	-0.013	-0.034
	(0.028)	(0.028)	(0.028)	(0.028)	(0.031)	(0.035)
Pro-trade (1-5 scale)	-0.000	0.000	-0.000	0.000	-0.000	-0.009
	(0.012)	(0.012)	(0.012)	(0.012)	(0.014)	(0.015)
Pro-immigration (1-5 scale)	-0.000	0.000	0.000	0.000	-0.000	-0.004
	(0.015)	(0.015)	(0.015)	(0.015)	(0.017)	(0.019)
Pro-tech. change (1-5 scale)	-0.000	-0.000	-0.000	-0.000	0.003	0.001
	(0.012)	(0.012)	(0.012)	(0.012)	(0.013)	(0.015)
Status $>$ Concrete (Pr. Component)	0.000	-0.000	-0.000	-0.000	-0.006	-0.005
	(0.012)	(0.012)	(0.012)	(0.012)	(0.013)	(0.015)
Observations	23,702	23,702	23,702	23,702	23,702	23,702

Robust standard errors in parenthesis. * $p < 0.10$, ** $p < 0.05$, *** $p < 0.01$. High I. Income: individual income treatment above the country monthly average. High C. Income: country income treatment above the country average. High income: household income above the country monthly average (3000 reals/euro, or 6000 USD). Very high income: monthly household income above 5000 reals/euro 5000, or 9000 USD). Machavellianism, narcissism and psychopathy are standardized to have mean zero and standard deviation of one, from the sum of scores in 5 questions. Status $>$ Concrete is the 2nd principal component of the responses to debriefing questions about income motivations in answering the conjoint, which features positive weights for status motivations for income and negative weights for material motivations for income.

Table S3. Covariate Balance Test for All Treatments, By Social Category. United States

	(1)	(2)	(3)	(4)	(5)	(6)
	E[Covariate | Treatment = 1] - E[Covariate | Treatment = 0]					
Variable	Democracy	Public Health	Effort	Equal Society	High I. Income	High C. Income
Fraction with High Income	-0.000	-0.000	-0.000	0.000	-0.005	-0.005
	(0.006)	(0.006)	(0.006)	(0.006)	(0.007)	(0.008)
Fraction with Very High Income	0.000	0.000	-0.000	0.000	-0.002	-0.000
	(0.004)	(0.004)	(0.004)	(0.004)	(0.005)	(0.005)
HH Wealth (0-10 index)	0.000	0.000	-0.000	0.000	0.014	0.003
	(0.030)	(0.030)	(0.030)	(0.030)	(0.034)	(0.038)
Parental Wealth (0-10 index)	0.000	0.000	-0.000	-0.000	-0.002	-0.009
	(0.031)	(0.031)	(0.031)	(0.031)	(0.035)	(0.039)
Fraction with college	0.000	0.000	0.000	-0.000	-0.001	-0.001
	(0.006)	(0.006)	(0.006)	(0.006)	(0.007)	(0.008)
Fraction with high school or less	0.000	-0.000	0.000	-0.000	0.001	0.003
	(0.003)	(0.003)	(0.003)	(0.003)	(0.004)	(0.004)
Age (continuous)	0.000	-0.000	0.000	0.000	0.149	0.311
	(0.222)	(0.222)	(0.222)	(0.222)	(0.248)	(0.277)
Fraction of females	-0.000	-0.000	-0.000	-0.000	-0.001	-0.001
	(0.006)	(0.006)	(0.006)	(0.006)	(0.007)	(0.008)
Machiavellianism (standardized index)	-0.000	-0.000	-0.000	0.000	0.002	-0.004
	(0.014)	(0.014)	(0.014)	(0.014)	(0.015)	(0.017)
Narcissism (standardized index)	0.000	0.000	-0.000	0.000	0.004	-0.001
	(0.014)	(0.014)	(0.014)	(0.014)	(0.016)	(0.018)
Psychopathy (standardized index)	-0.000	-0.000	0.000	0.000	-0.007	-0.009
	(0.014)	(0.014)	(0.014)	(0.014)	(0.016)	(0.017)
Right wing (1-10 scale)	-0.000	0.000	-0.000	0.000	-0.008	0.008
	(0.032)	(0.032)	(0.032)	(0.032)	(0.036)	(0.040)
Pro-trade (1-5 scale)	0.000	-0.000	0.000	-0.000	0.006	0.009
	(0.011)	(0.011)	(0.011)	(0.011)	(0.013)	(0.014)
Pro-immigration (1-5 scale)	-0.000	0.000	0.000	-0.000	0.002	-0.004
	(0.015)	(0.015)	(0.015)	(0.015)	(0.016)	(0.018)
Pro-tech. change (1-5 scale)	0.000	0.000	-0.000	0.000	0.006	0.009
	(0.011)	(0.011)	(0.011)	(0.011)	(0.012)	(0.014)
Status $>$ Concrete (Pr. Component)	-0.000	-0.000	0.000	-0.000	-0.003	0.006
	(0.015)	(0.015)	(0.015)	(0.015)	(0.017)	(0.019)
Observations	22,736	22,736	22,736	22,736	22,736	22,736

Robust standard errors in parenthesis. * $p < 0.10$, ** $p < 0.05$, *** $p < 0.01$. High I. Income: individual income treatment above the country monthly average. High C. Income: country income treatment above the country average. High income: household income above the country monthly average (3000 reals/euro, or 6000 USD). Very high income: monthly household income above 5000 reals/euro 5000, or 9000 USD). Machavellianism, narcissism and psychopathy are standardized to have mean zero and standard deviation of one, from the sum of scores in 5 questions. Status $>$ Concrete is the 2nd principal component of the responses to debriefing questions about income motivations in answering the conjoint, which features positive weights for status motivations for income and negative weights for material motivations for income.

Table S4. Willingness to Pay (using choice estimations)

	WTP for normalized income			WTP and income in $1,000 PPP		
	(1)	(2)	(3)	(4)	(5)	(6)
	Brazil	France	US	Brazil	France	US
Democracy	324.7***	233.6***	439.6***	4.128***	9.600***	26.38***
	(87.60)	(52.06)	(132.3)	(1.114)	(2.139)	(7.940)
Public Health	114.6***	203.7***	116.4**	1.457***	8.371***	6.982**
	(31.22)	(45.29)	(35.97)	(0.397)	(1.861)	(2.158)
Effort matters	26.40**	49.06***	65.11**	0.336**	2.016***	3.906**
	(9.749)	(11.69)	(20.94)	(0.124)	(0.480)	(1.256)
More Equal Society	21.94*	45.25***	-6.359	0.279*	1.859***	-0.382
	(9.114)	(11.47)	(8.059)	(0.116)	(0.471)	(0.484)
10% increase in Country Income	8.246***	-1.912	1.253	0.105***	-0.0786	0.0752
	(2.494)	(1.092)	(1.556)	(0.0317)	(0.0449)	(0.0933)
N	26740	23702	22736	26740	23702	22736
Measure of preference	Choice	Choice	Choice	Choice	Choice	Choice

Entries are ratios of AMCEs. Delta Method standard errors in parenthesis. ACMEs estimated with individual-level pair fixed effects and position controls. Columns 1-3: WTP as a percentage of the average income. Columns 4-6: WTP in thousands of PPP$. * $p < 0.10$, ** $p < 0.05$, *** $p < 0.01$.

Table S5. Estimation of Attributes' Weights (using choice estimations)

	(1)	(2)	(3)
	Brazil	France	US
Democracy	0.485***	0.359***	0.594***
	(0.0249)	(0.0168)	(0.0301)
Public Health	0.171***	0.313***	0.157***
	(0.0122)	(0.0150)	(0.0126)
Effort matters	0.0394***	0.0754***	0.0880***
	(0.00983)	(0.00755)	(0.0107)
More Equal Society	0.0327**	0.0695***	0.00859
	(0.00998)	(0.00853)	(0.0103)
Country Income (100% increase)	0.123***	0.0294	0.0169
	(0.0173)	(0.0153)	(0.0200)
Individual Income (100% Increase)	0.149***	0.154***	0.135***
	(0.0341)	(0.0287)	(0.0354)
N	26740	23702	22736
Measure of preference	Choice	Choice	Choice

Entries are ratios of AMCEs absolute values over the sum of all AMCEs absolute values. Delta Method standard errors in parenthesis. * $p < 0.10$, ** $p < 0.05$, *** $p < 0.01$. AMCEs specification includes individual-specific pair fixed effects and position controls.

Table S6. Means of covariates across groups defined by their individual weight of democracy and covariate differences across groups, Brazil

	(1)	(2)	(3)	(4)	(5)
	Means across democracy weight groups			Differences across groups	
Variable	Non-Dem†	Moderate Pro-Dem‡	Pro-Dem§	Moderate vs. Non	Moderate vs. Pro
Fraction with High Income	0.371	0.391	0.494	0.020	-0.094***
	(0.483)	(0.489)	(0.500)	(0.032)	(0.029)
Fraction with Very High Income	0.106	0.108	0.193	0.002	-0.074***
	(0.308)	(0.310)	(0.395)	(0.021)	(0.022)
HH Wealth (0-10 index)	3.957	3.744	4.114	-0.213	0.038
	(2.568)	(2.472)	(2.330)	(0.169)	(0.138)
Parental Wealth (0-10 index)	3.798	3.659	3.899	-0.139	0.106
	(2.636)	(2.521)	(2.408)	(0.173)	(0.141)
Fraction with college	0.114	0.163	0.240	0.049**	-0.069***
	(0.318)	(0.370)	(0.427)	(0.023)	(0.024)
Fraction with high school or less	0.465	0.414	0.289	-0.051	0.128***
	(0.499)	(0.493)	(0.453)	(0.033)	(0.027)
Age (continuous)	41.176	40.068	40.291	-1.109	-2.083**
	(15.417)	(15.200)	(15.421)	(1.024)	(0.894)
Fraction of females	0.512	0.539	0.497	0.027	0.046
	(0.500)	(0.499)	(0.500)	(0.033)	(0.029)
Machiavellianism (standardized index)	0.321	0.251	0.177	-0.070	0.131**
	(0.924)	(0.988)	(1.030)	(0.064)	(0.059)
Narcissism (standardized index)	0.286	0.267	0.209	-0.020	0.173***
	(0.909)	(0.977)	(0.967)	(0.063)	(0.055)
Psychopathy (standardized index)	-0.167	-0.158	-0.334	0.009	0.097*
	(1.006)	(1.009)	(0.932)	(0.067)	(0.056)
Right wing (1-10 scale)	5.661	5.764	5.619	0.104	-0.024
	(2.536)	(2.608)	(2.616)	(0.172)	(0.151)
Pro-trade (1-5 scale)	3.704	3.867	3.959	0.163***	-0.111**
	(0.867)	(0.827)	(0.855)	(0.057)	(0.049)
Pro-immigration (1-5 scale)	3.180	3.211	3.306	0.030	-0.089*
	(0.892)	(0.917)	(0.900)	(0.060)	(0.051)
Pro-tech. change (1-5 scale)	3.916	3.955	4.103	0.039	-0.145***
	(0.866)	(0.810)	(0.770)	(0.056)	(0.046)
Status $>$ Concrete (Pr. Component)	0.139	0.127	0.005	-0.011	0.003
	(1.186)	(1.148)	(1.106)	(0.078)	(0.063)
Observations	510	399	1,001	909	1,381

Columns 1 to 3 report mean values of covariates and standard deviations in parenthesis, splitting survey participants based on their individual weight of democracy, measured from ratings. † Non-Dem: negative or zero weight. ‡ Moderate Pro-Dem: positive but below or equal to the median weight. § Pro-Dem: positive and above the median. Columns 4 and 5 report differences between columns 1 and 2 and between columns 2 and 3, respectively. Standard errors are in parenthesis and * $p < 0.10$, ** $p < 0.05$, ***. High income: household income above the country monthly average (3000 reals/euro, or 6000 USD). Very high income: monthly household income above 5000 reals/euro 5000, or 9000 USD). Machavellianism, narcissism and psychopathy are standardized to have mean zero and standard deviation of one, from the sum of scores in 5 questions. Status $>$ Concrete is the 2nd principal component of the responses to debriefing questions about income motivations in answering the conjoint, which features positive weights for status motivations for income and negative weights for material motivations for income.

Table S7. Means of covariates across groups defined by their individual weight of democracy and covariate differences across groups, France

	(1)	(2)	(3)	(4)	(5)
	Means across democracy weight groups			Differences across groups	
Variable	Non-Dem†	Moderate Pro-Dem‡	Pro-Dem§	Moderate vs. Non	Moderate vs. Pro
Fraction with High Income	0.331	0.337	0.341	0.006	0.015
	(0.471)	(0.473)	(0.474)	(0.033)	(0.031)
Fraction with Very High Income	0.045	0.052	0.041	0.007	0.032**
	(0.209)	(0.223)	(0.199)	(0.015)	(0.013)
HH Wealth (0-10 index)	4.465	4.378	4.348	-0.086	0.180
	(2.033)	(1.943)	(1.840)	(0.139)	(0.122)
Parental Wealth (0-10 index)	4.147	4.088	4.115	-0.058	-0.013
	(2.129)	(2.158)	(2.066)	(0.149)	(0.137)
Fraction with college	0.397	0.412	0.430	0.015	-0.042
	(0.490)	(0.493)	(0.495)	(0.034)	(0.033)
Fraction with high school or less	0.186	0.166	0.130	-0.020	0.110***
	(0.389)	(0.372)	(0.336)	(0.027)	(0.023)
Age (continuous)	49.444	48.177	49.209	-1.267	1.192
	(17.081)	(16.722)	(17.862)	(1.176)	(1.152)
Fraction of females	0.481	0.530	0.527	0.049	0.011
	(0.500)	(0.500)	(0.500)	(0.035)	(0.033)
Machiavellianism (standardized index)	-0.036	0.044	-0.123	0.079	0.017
	(0.888)	(1.013)	(0.997)	(0.066)	(0.065)
Narcissism (standardized index)	-0.078	-0.108	-0.197	-0.030	0.030
	(0.911)	(0.960)	(0.977)	(0.065)	(0.064)
Psychopathy (standardized index)	0.278	0.210	0.068	-0.068	0.113*
	(0.883)	(0.859)	(0.884)	(0.061)	(0.058)
Right wing (1-10 scale)	5.519	5.470	5.434	-0.049	0.189
	(2.259)	(2.090)	(2.144)	(0.152)	(0.141)
Pro-trade (1-5 scale)	3.322	3.423	3.401	0.100	-0.048
	(0.901)	(0.977)	(0.946)	(0.065)	(0.064)
Pro-immigration (1-5 scale)	2.591	2.550	2.655	-0.041	-0.159**
	(1.191)	(1.176)	(1.159)	(0.082)	(0.077)
Pro-tech. change (1-5 scale)	3.397	3.459	3.421	0.062	0.029
	(0.912)	(0.938)	(0.881)	(0.064)	(0.059)
Status $>$ Concrete (Pr. Component)	0.174	0.035	-0.093	-0.139**	0.233***
	(0.884)	(0.942)	(0.882)	(0.063)	(0.059)
Observations	484	362	847	846	1,165

Columns 1 to 3 report mean values of covariates and standard deviations in parenthesis, splitting survey participants based on their individual weight of democracy, measured from ratings. † Non-Dem: negative or zero weight. ‡ Moderate Pro-Dem: positive but below or equal to the median weight. § Pro-Dem: positive and above the median. Columns 4 and 5 report differences between columns 1 and 2 and between columns 2 and 3, respectively. Standard errors are in parenthesis and * $p < 0.10$, ** $p < 0.05$, ***. High income: household income above the country monthly average (3000 reals/euro, or 6000 USD). Very high income: monthly household income above 5000 reals/euro 5000, or 9000 USD). Machavellianism, narcissism and psychopathy are standardized to have mean zero and standard deviation of one, from the sum of scores in 5 questions. Status $>$ Concrete is the 2nd principal component of the responses to debriefing questions about income motivations in answering the conjoint, which features positive weights for status motivations for income and negative weights for material motivations for income.

Table S8. Means of covariates across groups defined by their individual weight of democracy and covariate differences across groups, United States

	(1)	(2)	(3)	(4)	(5)
	Means across democracy weight groups			Differences across groups	
Variable	Non-Dem†	Moderate Pro-Dem‡	Pro-Dem§	Moderate vs. Non	Moderate vs. Pro
Fraction with High Income	0.326	0.277	0.315	-0.049	-0.020
	(0.469)	(0.448)	(0.465)	(0.033)	(0.031)
Fraction with Very High Income	0.124	0.110	0.108	-0.014	0.000
	(0.330)	(0.313)	(0.311)	(0.023)	(0.021)
HH Wealth (0-10 index)	4.426	4.439	4.250	0.013	-0.003
	(2.434)	(2.275)	(2.169)	(0.170)	(0.146)
Parental Wealth (0-10 index)	4.246	4.229	4.115	-0.017	0.083
	(2.517)	(2.451)	(2.214)	(0.178)	(0.153)
Fraction with college	0.306	0.320	0.357	0.014	-0.103***
	(0.461)	(0.467)	(0.479)	(0.033)	(0.032)
Fraction with high school or less	0.101	0.079	0.055	-0.022	0.038**
	(0.302)	(0.271)	(0.229)	(0.021)	(0.016)
Age (continuous)	47.973	49.933	53.685	1.960*	-5.324***
	(16.133)	(16.402)	(16.893)	(1.162)	(1.104)
Fraction of females	0.614	0.704	0.611	0.091***	0.026
	(0.487)	(0.457)	(0.488)	(0.034)	(0.032)
Machiavellianism (standardized index)	-0.171	-0.191	-0.387	-0.020	0.232***
	(1.009)	(0.979)	(1.032)	(0.071)	(0.067)
Narcissism (standardized index)	-0.158	-0.124	-0.273	0.034	0.104
	(1.019)	(1.064)	(1.113)	(0.074)	(0.071)
Psychopathy (standardized index)	-0.067	-0.123	-0.284	-0.056	0.299***
	(1.061)	(1.095)	(1.019)	(0.077)	(0.069)
Right wing (1-10 scale)	5.816	5.655	5.825	-0.161	-0.065
	(2.347)	(2.218)	(2.547)	(0.164)	(0.165)
Pro-trade (1-5 scale)	3.680	3.625	3.867	-0.055	-0.153***
	(0.889)	(0.837)	(0.810)	(0.062)	(0.053)
Pro-immigration (1-5 scale)	3.320	3.229	3.378	-0.092	-0.115
	(1.080)	(1.089)	(1.118)	(0.078)	(0.073)
Pro-tech. change (1-5 scale)	3.762	3.729	3.857	-0.034	-0.000
	(0.846)	(0.822)	(0.820)	(0.060)	(0.054)
Status $>$ Concrete (Pr. Component)	-0.117	-0.249	-0.577	-0.132	0.382***
	(1.178)	(1.109)	(1.147)	(0.082)	(0.076)
Observations	484	328	812	812	1,216

Columns 1 to 3 report mean values of covariates and standard deviations in parenthesis, splitting survey participants based on their individual weight of democracy, measured from ratings. † Non-Dem: negative or zero weight. ‡ Moderate Pro-Dem: positive but below or equal to the median weight. § Pro-Dem: positive and above the median. Columns 4 and 5 report differences between columns 1 and 2 and between columns 2 and 3, respectively. Standard errors are in parenthesis and * $p < 0.10$, ** $p < 0.05$, ***. High income: household income above the country monthly average (3000 reals/euro, or 6000 USD). Very high income: monthly household income above 5000 reals/euro 5000, or 9000 USD). Machavellianism, narcissism and psychopathy are standardized to have mean zero and standard deviation of one, from the sum of scores in 5 questions. Status $>$ Concrete is the 2nd principal component of the responses to debriefing questions about income motivations in answering the conjoint, which features positive weights for status motivations for income and negative weights for material motivations for income.

4. The Meaning of Democracy

As in any other conjoint study, the interpretation of any AMCE could be affected by the possibility that respondents associate an attribute of the treatment with another attribute that is omitted from our experimental design. In the case of democratic elections, the respondents' preferences may be driven by an intrinsic desire to have democracy understood as a procedure to select governments according to principles of fairness and equal participation – the way in which we have constructed the attribute in our surveys. But it may also be the case that respondents support democracy because of some expected consequences from having democratic elections. For instance, individuals may believe that democracy is good because it leads to more redistribution, prosperity, or equality of opportunity.

To approximate the intrinsic preference for democracy, our choice experiment holds constant an important vector of characteristics which may themselves be affected (or believed to be affected) by democracy. As a result, we interpret the AMCE from this conjoint experiment as referring to the preference for democracy net of its effects on individual and country prosperity, public health, the importance of effort, and economic inequality, which are held constant. While there may be other consequences of democracy that individuals may have in mind, this is a rather comprehensive list, and hence, we take the AMCE from this conjoint experiment to be fairly close to an intrinsic preference for democracy.

In addition, our results show that even when jointly evaluating democratic elections and other relevant social attributes that may be associated with it, democracy remains as the most important attribute to respondents. This suggests that the difference between the AMCE we estimate for democracy and an AMCE that would, in addition, encompass all the indirect effects of democracy (i.e., because of extrapolating how a democratic society would look like in terms of institutions and prosperity) is unlikely to be large. To show this to be the case, consider a hypothetical scenario according to which democratic elections would increase by 10% the probability of having public health insurance, a more equal society, and a society where effort matters more than connections, and that they would also lead to a 10% increase in expected individual and country income. Employing the estimates of each one of these separate attributes and adding the impact of all the changes in this scenario to the original AMCE for democracy, the total impact of democracy would rise from our current AMCE estimate for democracy of 1.27 to 1.35 for Brazil, 1.01 to 1.13 for France, and 1.51 to 1.58 for the US (AMCEs measuring preferences via ratings, with normalized income and pair fixed effects); or from 0.403 to 0.426 (Brazil), 0.373 to 0.422 (France), or 0.497 to 0.518 (US), when measuring preferences via choices, using the same specification.* The total estimated impact would increase because democratic elections tend to be positively correlated with all the attributes that feature positive AMCEs and that we are boosting in this scenario. The change from the original AMCE is, however, small.

* Formally, we compute: $\beta^{Democracy}_{Total} = \beta^{Democracy}_{AsProcedure} + \beta^{OtherOutcomes} \times (E[OtherOutcomes|Democracy = 1] - E[OtherOutcomes|Democracy = 0])$. We assume an associated 10 percent increase for each attribute due to democracy and with "Other Outcomes" referring to the attributes of individual income, country income, public health, effort and equal society we control for in the conjoint
